# Assessing alexithymia: the proposal of a psychometric tool based on spheric videos

**DOI:** 10.3389/fnhum.2024.1375342

**Published:** 2024-03-18

**Authors:** Gloria Simoncini, Francesca Borghesi, Valentina Mancuso, Elisa Pedroli, Pietro Cipresso

**Affiliations:** ^1^Department of Psychology, University of Turin, Turin, Italy; ^2^Faculty of Psychology, eCampus University, Novedrate, Italy; ^3^Department of Geriatrics and Cardiovascular Medicine, IRCCS Istituto Auxologico Italiano, Milan, Italy

**Keywords:** alexithymia, assessment, 360° videos, psychometrics, emotion, neuroscience

## Abstract

The present perspective introduces a novel psychometric tool designed to enhance the evaluation of alexithymia. Alexithymia, a condition marked by difficulties in recognizing and expressing emotions, along with a propensity to direct attention outside rather than toward one’s own interior experiences, is commonly investigated through self-report questionnaires. These instruments assume that individuals have sufficient self-awareness and abstraction capabilities, which restricts the understanding of the underlying mechanisms of emotional recognition in individuals who do not possess these capacities. To address this lack, emerging technologies like virtual reality (VR) and 360° videos facilitate the recreation of immersive contexts, enabling subjects to engage with scenarios even remotely. Our innovative tool employs spherical video technology to recreate social and non-social scenarios that elicit emotions. Psychophysiological measures are collected during video observation; then, questions are asked to investigate how the subject consciously processes the emotions they experienced. This multimodal approach aims to capture both implicit and explicit emotion processing, providing a comprehensive assessment. Overall, the proposed psychometric tool offers the potential for a more nuanced understanding of alexithymic traits and their real-life impact, empowering clinicians to tailor treatment processes to individual needs based on a richer set of information.

## Introduction

Alexithymia refers to a condition where individuals are unable to recognize, explicitly define, and articulate their own emotions (Nemiah, 1977; Hogeveen and Grafman, 2021), although, at an implicit psychophysiological level, variations can be observed (Panayiotou et al., 2018).

Self-reporting is a commonly used method to study alexithymia, providing information about its characteristics and connections to psychological and neurological conditions (Luminet et al., 2018). However, there are concerns that self-report assessments may not accurately capture contextualized behavioral tendencies (Buchanan, 2016), as they can be influenced by subjects’ self-judgment, self-consciousness, and generalization processes (Wilson and Dunn, 2004; Averbeck, et al., 2013). Relying solely on self-report tools leads to a huge gap in the understanding of the neural and behavioral mechanisms underlying emotional awareness and its breakdown in cases of alexithymia. In addition, self-report questionnaires provide a picture detached from the situational and social level in which alexithymia manifests itself.

In the diagnostic phase it is necessary to have a snapshot, albeit initial, of the individual’s functioning in relation to contextual stimuli; but how can this be observed in the clinical environment? One challenge might be recreating the complex psychological and emotional experiences that individuals encounter in their daily lives in a clinical setting. Technologies like virtual reality (VR) are valuable in recreating specific environments and situations that can evoke emotions and feelings, enabling researchers and clinicians to observe and analyze a subject’s behavior during these conditions (Mancuso et al., 2023). Another crucial point concerns the practicality of a particular instrument in a clinical setting; assessment tools must be quick and simple to administer and correct, while at the same time providing a comprehensive picture about a particular condition.

To address all these needs, this paper proposes the development of an alternative method for assessing alexithymia that exploits 360° video technology. This particular form of technology is specifically designed for immersive viewing through VR headsets or on conventional devices (Borghesi et al., 2022). Despite not being able to directly interact with the narrative, users develop their own sense of the story/situation by selecting where to look, which gives them “agency” over the narrative. These spherical videos provide a chance for individuals to be fully engrossed in genuine natural settings (Borgnis et al., 2021). As compared to VR, 360° videos overcome the costs’ issue, while guaranteeing the presence of ecological environments.

Employing 360° videos as an assessment tool for alexithymia might offer a more direct understanding of an individual’s abilities to recognize and describe his own feelings when faced with emotional scenarios, allowing the clinician to detect specific strengths and weaknesses to guide the subsequent treatment. In the present perspective we will propose a new ecological and practical approach for alexithymia assessment that takes advantage of 360° video technology.

## Alexithymia

As salient internal states, emotions can promote the subject’s adaptive behavior toward various stimuli (Russell, 2003; Barrett et al., 2007). Emotions aid in responding effectively to the environment, such as evading danger or displaying rivalry, thus allowing individuals to respond flexibly based on their experiences (Damasio, 2004; LeDoux, 2012). The malfunctioning of these mechanisms is the basis of conditions such as autism spectrum disorders and alexithymia (Hogeveen and Grafman, 2021).

The emotional process can be subdivided into implicit valence and arousal signals that are generated primarily via subcortical and autonomic circuits, and a more complex explicit dimension primarily computed via cortical circuits (LeDoux and Pine, 2016). However, the neural circuits necessary for generating emotional awareness remain unclear and there is a lack of consensus on the fundamental question of whether emotional awareness represents an epiphenomenon or a functional dimension that mobilizes adaptive cognitive-behavioral responses to felt emotions (Adolphs et al., 2019).

In the current article, we focus on alexithymia, a condition characterized by an impaired ability to recognize, label, and describe one’s feelings (Nemiah, 1977). Individuals with alexithymia exhibit a dissociation between implicit and explicit emotional responses (Nielson and Meltzer, 2009; Gaigg et al., 2018). Notably, this trait is considered a dimensional attribute with a typical distribution in the general population (Parker et al., 2008). Additionally, studies (Duddu et al., 2003) have substantiated heightened alexithymia levels in psychosomatic patients, identifying it as a transdiagnostic risk factor for various psychopathologies, including depression (Honkalampi et al., 2001), anxiety disorders (Zeitlin and McNally, 1993), personality disorders (Berenbaum, 1996), eating disorders (Taylor et al., 1996), and substance use (Thorberg et al., 2009).

### Theoretical components of alexithymia assessment

The primary development of the alexithymia concept has been carried out by two research groups: the Toronto and Amsterdam groups. The Toronto group (Taylor et al., 1999) developed a comprehensive model of alexithymia, drawing from both psychoanalytic concepts and cognitive theories of emotion processing [e.g., Bucci (1997) *multiple code theory*, and Lane and Schwartz’s (1987) *cognitive-developmental theory of levels of emotional awareness*]. They identified four interconnected components: difficulty identifying feelings in oneself (DIF), difficulty describing feelings (DDF), an externally oriented thinking (EOT) style fixated on external details, and limited imaginative processes (difficulty fantasizing; DFAN). The Toronto model has become the prevailing definition of alexithymia in current literature.

The Amsterdam group, led by Vorst and Bermond (2001), presented an alternative perspective on alexithymia. They built upon the four components of alexithymia introduced by the Toronto group (DIF, DDF, EOT, and DFAN) and added a fifth component called “reduced emotional reactivity” (or difficulty emotionalizing; DEMO). This concept refers to the extent to which an individual responds emotionally to events that trigger emotions (Vorst and Bermond, 2001).

Hence, empirical studies seem to endorse the majority of the statements within the Toronto (Bagby et al., 1994) and Amsterdam models (Vorst and Bermond, 2001). Both models concur that DIF, DDF, and EOT are interconnected facets of an underlying shared concept, and this is backed by robust empirical evidence. Nevertheless, the existing body of empirical research indicates that certain aspects of these models might be inaccurately defined (Preece et al., 2017).

Preece et al. (2017) attention-appraisal model of alexithymia integrates Gross (2015) emotion regulation model and Lane and Schwartz (1987) cognitive-developmental theory of emotional awareness. Gross’s model posits that emotions involve four stages: situation, attention, appraisal, and response, with emotions arising from how individuals value stimuli. The attention-appraisal model aligns alexithymia with Gross’s framework, linking Externally Oriented Thinking (EOT) to attention challenges and DIF and DDF to appraisal issues (Preece et al., 2017, 2024).

**Figure 1:**
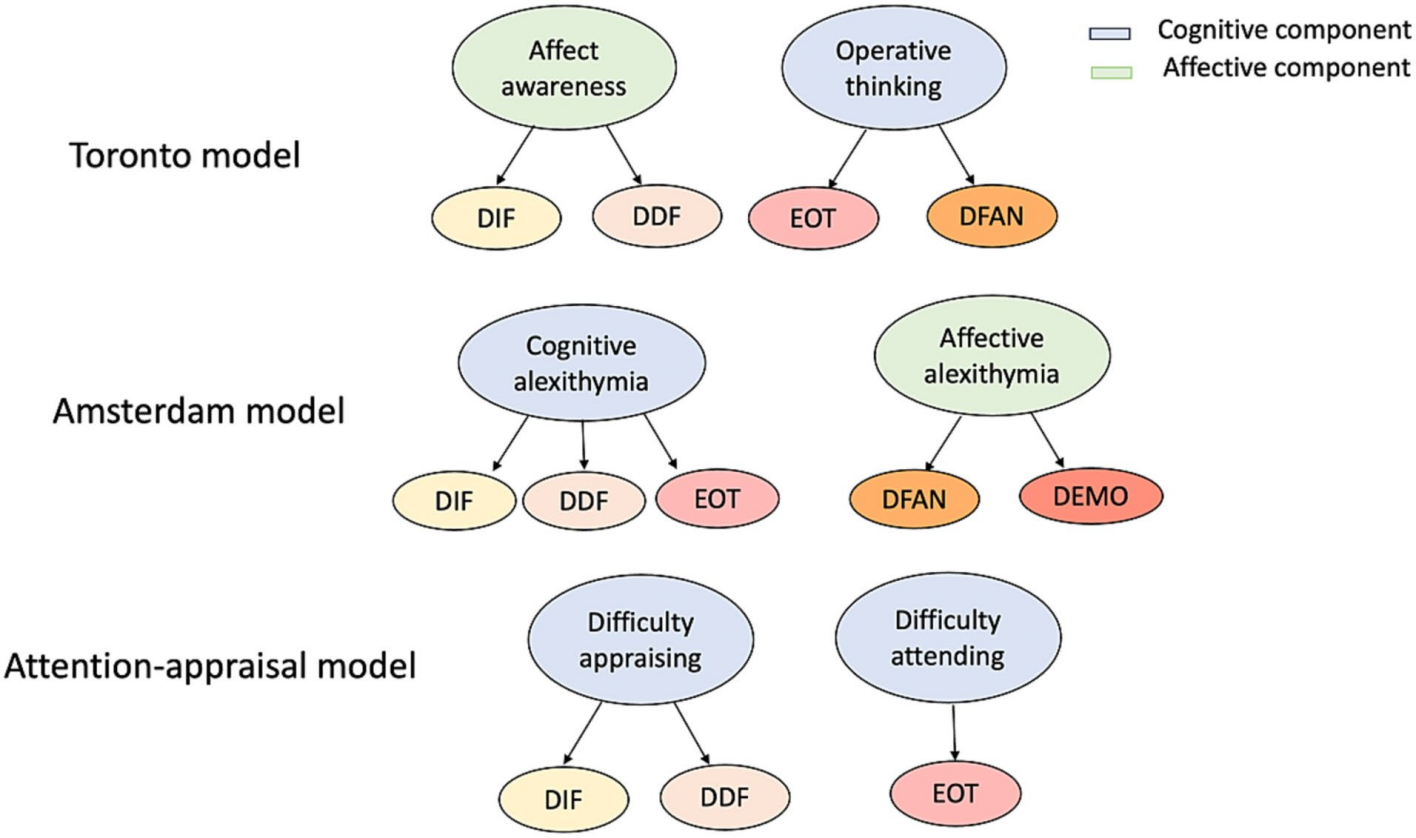
A graphical depiction of the three models mentioned above with the different components and sub-components, adapted from Preece et al. (2017).

Additionally, the model incorporates Lane and Schwartz’s (1987) cognitive-developmental theory, which describes five developmental levels to explain how individuals understand and experience emotion. Higher levels denote refined emotional understanding, contrasting with the underdeveloped emotion schemas observed in individuals with elevated alexithymia levels. This deficiency, evident in poor organization, differentiation, and integration (Lane et al., 1996; Lundh et al., 2002; Suslow and Junghanns, 2002; Luminet et al., 2006; Vermeulen et al., 2006), impairs the focus on relevant emotional elements in the attention phase and the interpretation of emotional information in the appraisal phase. Consequently, individuals with high alexithymia struggle to effectively assess emotions at specific levels (Preece et al., 2017, 2024; Figure 1).

In the present perspective all three theoretical models will be considered, trying to integrate the different contributions in the development of the new assessment tool.

### The main assessment tools for alexithymia

The Toronto team developed two assessment tools for alexithymia based on their model: the 20-item Toronto Alexithymia Scale (TAS-20) (Bagby et al., 1994) and the Toronto Structured Interview for Alexithymia (TSIA) (Bagby et al., 2006). The TSIA is an evaluator-rated tool covering DIF, DDF, EOT, and DFAN, while the TAS-20 is a self-report survey focusing on DIF, DDF, and EOT.

The Amsterdam group created the Bermond-Vorst Alexithymia Questionnaire (BVAQ) (Vorst and Bermond, 2001), a self-report evaluation that aligns with their model. It assesses DIF, DDF, EOT, DFAN, and DEMO. The correlations between the BVAQ DEMO items and other constructs have shown variability in their directions (Watters et al., 2016), and in some sample groups, the DEMO subscale exhibited inadequate internal consistency (Müller et al., 2004).

**Figure 2:**
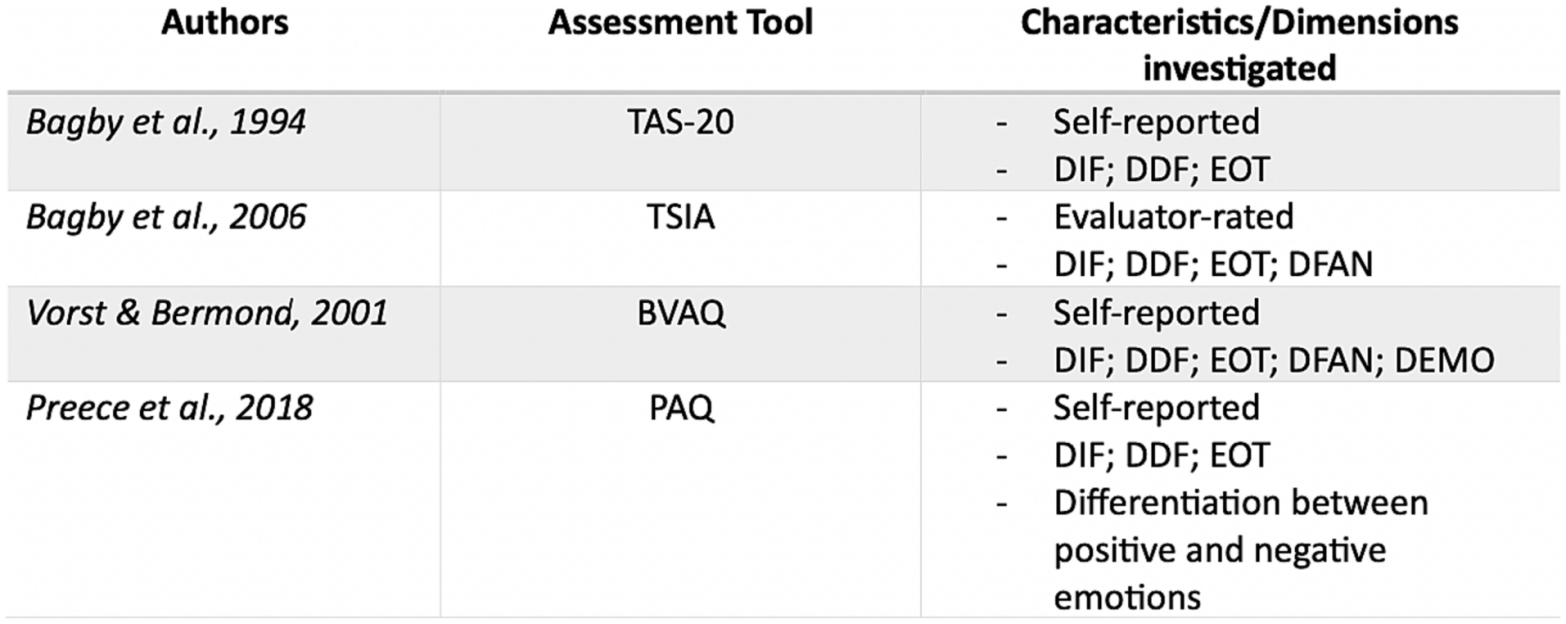
Summary image of the measuring instruments mentioned above.

Preece et al. (2018) developed the Perth Alexithymia Questionnaire (PAQ), a 24-item self-report assessment tool designed to evaluate alexithymia within the attention-appraisal model framework. It adheres to three specific measurement criteria: DIF, DDF, and EOT. The PAQ’s items related to the appraisal stage consider emotional valence, assessing an individual’s ability to appraise both negative and positive emotions. The PAQ demonstrated high internal consistency reliability for its subscale and composite scores and proved effective in assessing alexithymia (Preece et al., 2018; Figure 2).

## 360° videos and their application for clinical assessment

Over the past decade, VR technology has significantly advanced the healthcare sector (Riva, 2005; Freeman et al., 2017; Kyaw et al., 2019; Riva et al., 2020), enhancing user-friendly and visually engaging settings for experimentation, clinical work, assessments, and rehabilitation (Cipresso et al., 2018). Nevertheless, despite its potential, VR also comes with technical and psychometric challenges. Firstly, most VR systems require a computer and external tracking devices, thus limiting the user’s movement and necessitating complex setups. Additionally, developing VR environments is often resource-intensive and costly, as each experiment requires customization (Borghesi et al., 2022). Secondly, there is a lack of literature on the test–retest reliability and usability of VR tools. Limited studies have examined applicability, showing that these instruments are generally usable, intuitive, engaging and safe, but more research is needed (Freeman et al., 2017; Borgnis et al., 2022).

To address some of these challenges, 360° videos have emerged as a potential solution. They consist of a series of images capturing a full 360-degree view, with specific time interval between them (Borghesi et al., 2022). 360° videos are designed to be viewable through VR headsets; however, they can also be enjoyed on flat-screen devices, such as phones or computers, by simply moving the viewpoint with a mouse or a finger. The subject cannot interact directly with the environment but has the possibility to choose where to focus his attention, structuring his personal understanding of the virtual context. The opportunity to create a sense of presence in the user is a feature that distinguishes 360° videos from 2D videos, giving the subject the feeling of being within the virtual environment. Consequently, 360° videos can evoke strong emotional responses, making users feel physically present and even giving them the illusion of interacting and reacting as if they were in the real world (Chirico et al., 2017; Ventura et al., 2019; Borghesi et al., 2022, 2023a).

Within any 360° videos, users have the option to adopt either a first-person or third-person perspective (Borghesi et al., 2022). Some recent studies (Higuera-Trujillo et al., 2017; Chirico and Gaggioli, 2019; Brivio et al., 2021) have shown that 360° videos are able to elicit similar psychological and physiological responses to those produced by the real physical environment. Specifically, it emerged that the virtual and natural environments generated the same emotional responses in terms of valence, although with reduced intensity (Chirico and Gaggioli, 2019).

Through 360° video technology, healthcare professionals can enhance ecological assessments, mitigating biases associated with self-reported measures. Constructing assessment tools with higher ecological validity addresses challenges stemming from subjects’ self-evaluation and self-awareness during examiner interaction (Parsons, 2015). Previous studies have exploited the potential of 360° videos to assess personality and cognitive functioning such as executive functions and memory (Cipresso and Riva, 2016; Realdon et al., 2019; Stramba-Badiale et al., 2020; Borgnis et al., 2021; Della Libera et al., 2023; Bruni et al., 2023a,b).

Cipresso and Riva (2016) introduced a novel approach that utilized immersive scenarios to evaluate personality traits. Realdon et al. (2019) used a 360° version of the Picture Interpretation Test (PIT) to detect executive dysfunction, showing promise in identifying issues in individuals with Multiple Sclerosis and Parkinson’s disease. Moreover, Borgnis et al. (2021) introduced EXIT 360°, an innovative tool designed to assess executive functions (EFs) in an engaging and enjoyable manner. This tool evaluates executive impairments affecting daily life and independence within real-world scenarios, aligning with the need for contextual EF assessments. Lastly, Della Libera et al. (2023) explored the utility of 360° immersive videos in evaluating common psychological symptoms in a naturalistic cafe setting. The study revealed that the immersive experience effectively triggered the emergence of these symptoms, and participants’ predispositions played a role in their experiences.

These contributions highlight that offer healthcare professionals a more ecologically valid assessment environment, enabling the collection of richer and more accurate data on various aspects of psychological and cognitive functioning.

## An initial presentation of a 360° tool for alexithymia assessment

The present perspective aims at proposing and discussing the design, creation and implementation of ALEX-360°, a 360° videos tool for an innovative and ecologically valid assessment of alexithymia.

As noted previously, although self-administered assessment measures attempt to provide a general picture of the person’s current situation, they are decontextualized. They also presuppose that the subject has good abstraction and awareness skills to answer questions as accurately as possible about his moods. However, the person with alexithymia has difficulties precisely in this, that is, in recognizing and describing his feelings. For this reason, the new approach that we intend to propose involves the creation of environmental, social and non-social contexts, in the form of 360° videos, with which the subject can come into contact.

The theoretical assumptions consider the three factors that characterize the construct of alexithymia: DIF, DDF, and EOT. Furthermore, the attention-appraisal model proposed by Preece et al. (2017) and the theory of emotional awareness by Lane and Schwartz (1987) are taken up again.

### 360° real environments

As scenarios, we propose twenty 360° short videos (duration of 15 s each) depicting social and non-social scenes capable of eliciting particular emotions. The use of 360° videos allows the subjects to immerse themself in a real situation from a first-person point of view (Borghesi et al., 2022). Even if they cannot interact with the environment, they can explore it by moving the mouse of the computer or the finger on the phone’s screen.

The 20 videos will consist of interpersonal and non-interpersonal scenes, 10 with positive valence (e.g., people having fun in the park; the vision of some paintings) and 10 with negative valence (e.g., a thief stealing a wallet; the arrival of a thunderstorm). This distinction will allow us to observe whether the subject experiences particular difficulties in recognizing and describing his emotions in situations with a particular valence (Preece et al., 2017).

### Structure of the assessment tool

**Figure 3:**
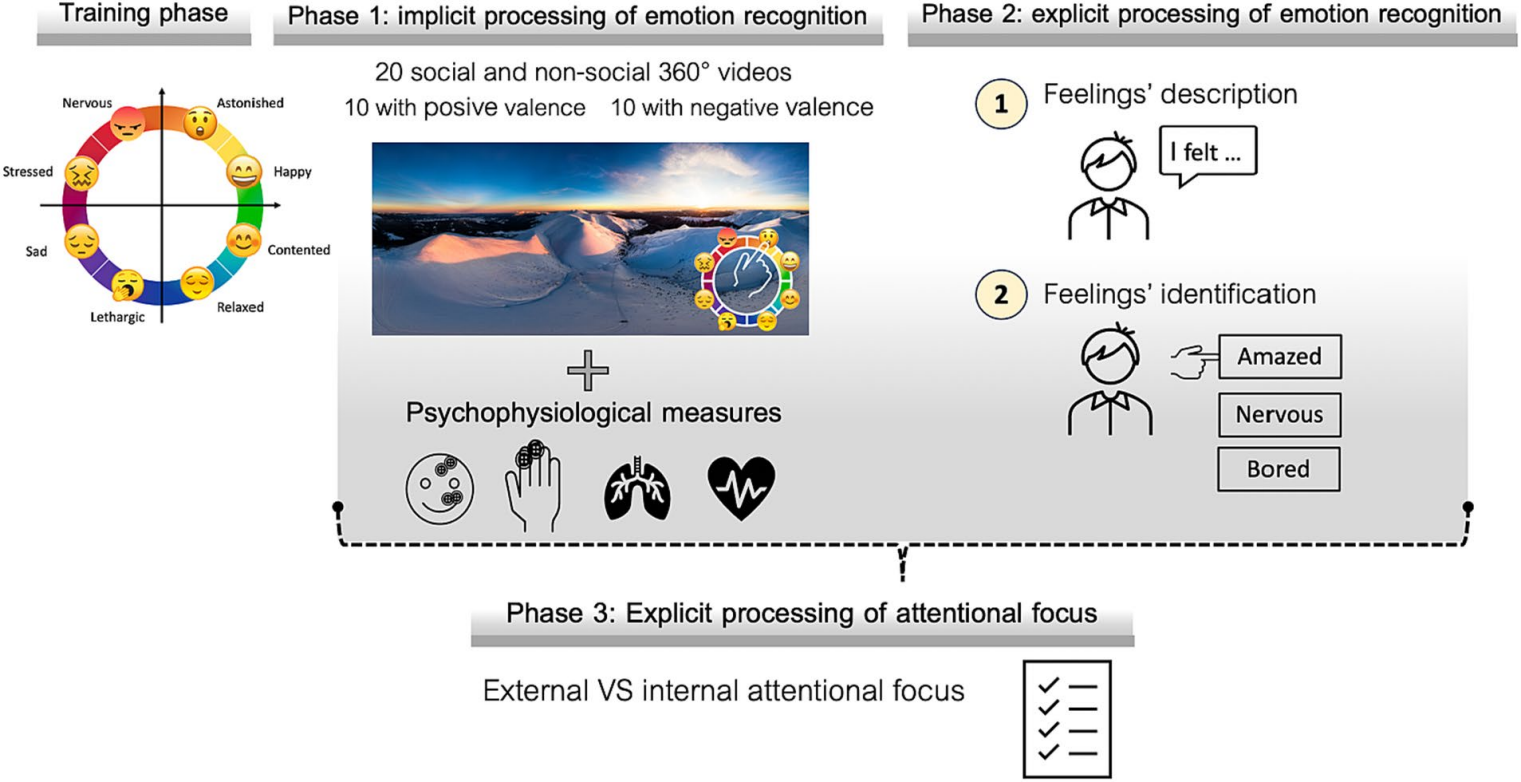
Different phases of the assessment procedure.

Before starting the test, the subject undergoes a training phase to familiarize himself with a circular “emotional wheel” (see Figure 3) presented with verbal labels.

During the observation of the 20 videos, displayed on a computer monitor, participants use the “emotional wheel” to select the emoji that best mirrors their current feelings, aligning with Russell’s core affect model (Russell, 2003). The chosen theoretical framework incorporates valence and arousal dimensions to capture nuanced emotional experiences. Utilizing eight emojis facilitates graphical emotional recognition without verbal labels, as emojis are established in digital communication with known emotional associations (Shoeb and de Melo, 2020; Toet et al., 2020). Positioned at the bottom right of the screen to avoid distraction (Zhang et al., 2020), the emotional wheel enables real-time assessment of the subject’s activation level and emotion value in a specific situation.

Due to its characteristics, ALEX-360° lends itself to the collection of physiological parameters, such as skin conductance (SC), heart rate variability (HRV), facial electromyographic movements (Mauri et al., 2010). Skin conductance will be measured using two electrodes placed on the ring and index fingers; heart rate variability will be determined via a photoplethysmograph to record the Blood Volume Pulse (BVP); facial electromyography (f-EMG) will be recorded using electrodes positioned on the zygomatic and corrugator muscles. The collection of physiological and behavioral data can provide a comprehensive information of the functioning of the subject.

After watching the video, subjects are asked to describe what they felt. In this phase, potential difficulties in describing feelings (DDF) at an explicit level can emerge. Based on the type of the answer, it is possible to establish the level of awareness (Lane and Schwartz, 1987; Lane and Smith, 2021) that the user has: bodily/visceral sensations, action tendency, pervasive emotions, differentiated emotions or richer differentiations of quality and intensity.

Following the description provided, the subject is asked to identify among three alternatives the one that best reflects his state of mind; here, it is possible to observe difficulties in identifying feelings (DIF). This must happen following the description given, in order not to influence the subjects’ response and direct them toward an alternative that they would not select of their own free will. In this phase, we can also detect any congruence between the description provided previously and the identification requested.

At the end of the test, some statements are presented with which subjects are asked to express their degree of agreement or disagreement. This conclusive step aims at exploring externally oriented thinking (EOT) by referring to the test just taken. The statements are: “I’m not used to paying attention to what I feel”; “I would have preferred to just watch the videos rather than focus on what I felt”; “The tasks presented were difficult for me.”

The scoring will be quantitative and will be determined by adding up the scores achieved in the different dimensions (DDF, DIF, and EOT). Through these stages, it will be possible to observe the ways in which the subject reacts and approaches the stimuli presented, both in real time and *a posteriori*. Although the tool presented contains questions to which the subject must answer personally, they refer to videos seen previously, thus providing a specific contextual response. This would overcome any problems related to the subject’s difficulties in generalization or abstraction. Moreover, the duration of the test, approximately 15–20 min, and the simplicity of administration make it practical to use in the clinical setting.

## Conclusion

This perspective endorses the proposal to develop a novel tool, ALEX-360°, designed to assess alexithymia. ALEX-360°, leveraging 360° videos as stimuli, shows promise in elucidating the underlying processes of alexithymia. Specifically, this tool enables the clinician to obtain additional insights into the subject’s functioning by observing behavior in a more ecologically valid context. Furthermore, ALEX-360° facilitates the identification of difficulties and the collection of physiological data, informing future treatment approaches.

Creating 360° videos *ad hoc* poses technical challenges that need to be addressed from the very beginning. A further potential limitation of this tool is its capacity to encompass and identify all aspects of alexithymia; it is uncertain if milder forms of alexithymia will be detected by the tool. In addition, laboratory experiments with both healthy subjects and individuals with alexithymia are required to confirm the efficiency of ALEX-360°.

A tool as ALEX-360° holds potential in shaping personalized and user-centered medicine. Regarding this guidance on using the tool will be offered to ensure it is user-friendly and clinician-friendly. Indeed, using artificial intelligence algorithms, the detection of alexithymic traits could be automated and personalized: based on a training dataset, the app could integrate automated scoring inherent to the areas most compromised in terms of recognition and emotional expression (Picard, 2003; Cipresso et al., 2021; Borghesi et al., 2023b).

The accessibility and clinical usability of ALEX-360° could redefine the evaluation process for individuals with alexithymia, delineating different severity levels and pinpointing the subjects’ emotional experiences. In other words, what they feel, how they feel. On the other hand, the nature of the test will involve the subject in a more dynamic activity compared to completing a self-report questionnaire, engaging his attention and interest for a relatively limited time, about 15 min. This feature is fundamental in the context of clinical and neuropsychological evaluation characterized by the administration of multiple tests; a long battery of tests can in fact influence not only the individual’s attention but also their motivation and collaboration. ALEX-360°, thanks to its strong engagement and dynamism, would solve this problem, engaging the patient in an almost playful activity.

In conclusion, ALEX-360°, with its capacity to gather informative data, emerges as a noteworthy and innovative aid in the diagnostic process, as well as in formulating treatment and rehabilitation strategies based on the patient’s unique resources.

## Data availability statement

The original contributions presented in the study are included in the article/supplementary material, further inquiries can be directed to the corresponding author.

## Author contributions

GS: Conceptualization, Methodology, Writing – original draft. FB: Conceptualization, Methodology, Writing – review & editing. VM: Writing – review & editing. EP: Writing – review & editing. PC: Conceptualization, Writing – review & editing.
